# Supplementary material for: Identification of photoperiod-induced specific miRNAs in the adrenal glands of Sunite sheep (Ovis aries)
Source: Front Vet Sci. 2022 Jul 22;9:888207. doi: 10.3389/fvets.2022.888207 (PMC9354845; doi:10.3389/fvets.2022.888207)
Supplement: Supplementary file 1 [file Data_Sheet_1.ZIP › Supplementary file/miRDeep2 file.zip!/miRDeep2 file/Know_Structure/SP42A2/oar-mir-409.pdf]

miRBase precursor : oar-mir-409
Total read count : 18568
oar-miR-409-5p read count : 3576
oar-miR-409-3p read count : 14987
remaining reads : 5

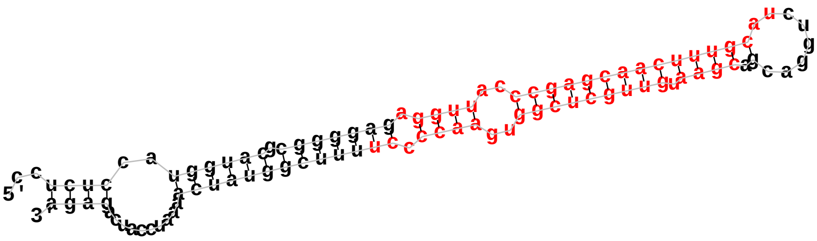

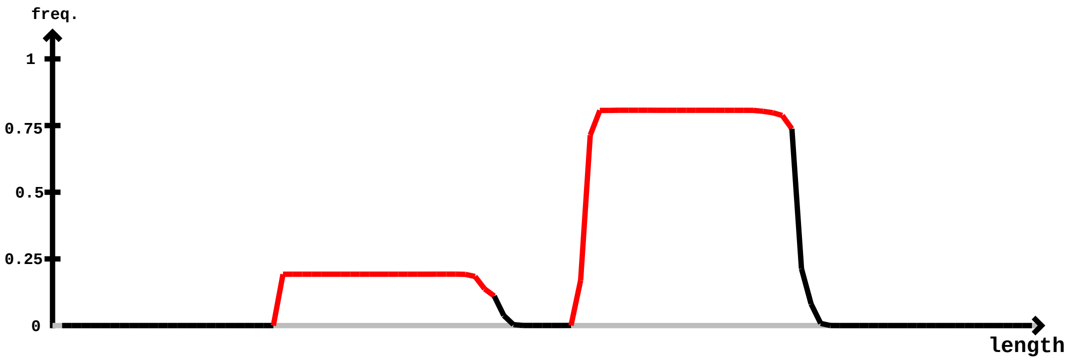


```
                                        oar-miR-409-5p
                                                                      oar-miR-409-3p
5'- ccucuccaugguacgcggggagagguuacccgagcaacuuugcaucuggacgacgaauguugcucggugaaccccuuuucgguaucaaaauccaucagaga   -3'    exp
    .((((...((((((.(((((((.((((..((((((((((((((........).)))).)))))))))..)))).)))))))))))))).........)))).    reads   mm    sample
    ......................agguuacccgagcaacu..............................................................    1       0     seq
    ......................agguuacccgagcaacuuu............................................................    14      0     seq
    ......................agguuacccgagcaacuuug...........................................................    146     0     seq
    ......................agguuacccgagcaacuuugc..........................................................    866     0     seq
    ......................agguuacccgagcaacuuugca.........................................................    495     0     seq
    ......................agguuacccgagcaacuuugcau........................................................    1358    0     seq
    ......................agguuacccgagcaacuuugcauc.......................................................    632     0     seq
    ......................agguuacccgagcaacuuugcaucu......................................................    58      0     seq
    ......................agguuacccgagcaacuuugcaucug.....................................................    2       0     seq
    .......................gguuacccgagcaacuuugc..........................................................    1       0     seq
    .......................gguuacccgagcaacuuugca.........................................................    1       0     seq
    .......................gguuacccgagcaacuuugcau........................................................    1       0     seq
    .......................gguuacccgagcaacuuugcauc.......................................................    1       0     seq
    ......................................uuugcaucuggacgacgaauguu........................................    3       0     seq
    .......................................uugcaucuggacgacgaauguu........................................    1       0     seq
    ............................................ucuggacgacgaauguugcucggu.................................    1       0     seq
    ...................................................gacgaauguugcucggugaacccc..........................    1       0     seq
    ....................................................acgaauguugcucggugaac.............................    3       0     seq
    ....................................................acgaauguugcucggugaaccccu.........................    1       0     seq
    .....................................................cgaauguugcucgguga...............................    1       0     seq
    .....................................................cgaauguugcucggugaa..............................    3       0     seq
    .....................................................cgaauguugcucggugaac.............................    43      0     seq
    .....................................................cgaauguugcucggugaacc............................    46      0     seq
    .....................................................cgaauguugcucggugaaccc...........................    73      0     seq
    .....................................................cgaauguugcucggugaacccc..........................    315     0     seq
    .....................................................cgaauguugcucggugaaccccu.........................    1935    0     seq
    .....................................................cgaauguugcucggugaaccccuu........................    533     0     seq
    .....................................................cgaauguugcucggugaaccccuuu.......................    163     0     seq
    .....................................................cgaauguugcucggugaaccccuuuu......................    30      0     seq
    .....................................................cgaauguugcucggugaaccccuuuuc.....................    3       0     seq
    ......................................................gaauguugcucggugaa..............................    1       0     seq
    ......................................................gaauguugcucggugaac.............................    19      0     seq
    ......................................................gaauguugcucggugaacc............................    48      0     seq
    ......................................................gaauguugcucggugaaccc...........................    103     0     seq
```

oar-miR-409-5p

oar-miR-409-3p

ccucuccaugguacgcggggagagguuacccgagcaacuuugcaucuggacgacgaauguugcucggugaaccccuuuucgguaucaaaauccaucagaga

....gaauguugcucggugaacccc..........................	536	0	seq
....gaauguugcucggugaaccccu.........................	6534	0	seq
....gaauguugcucggugaaccccuu........................	1729	0	seq
....gaauguugcucggugaaccccuuu.......................	1038	0	seq
....gaauguugcucggugaaccccuuuu......................	93	0	seq
....gaauguugcucggugaaccccuuuuc.....................	1	0	seq
....aauguugcucggugaacc............................	2	0	seq
....aauguugcucggugaaccc...........................	17	0	seq
....aauguugcucggugaacccc..........................	75	0	seq
....aauguugcucggugaaccccu.........................	1273	0	seq
....aauguugcucggugaaccccuu........................	189	0	seq
....aauguugcucggugaaccccuuu.......................	143	0	seq
....aauguugcucggugaaccccuuuu......................	23	0	seq
....auguugcucggugaaccccuu........................	1	0	seq
....uguugcucggugaaccccuuu.......................	10	0	seq
....uguugcucggugaaccccuuuu......................	1	0	seq
....guugcucggugaaccccuuu.......................	1	0	seq
